# Activation of Pregnane X Receptor by Pregnenolone 16 α-carbonitrile Prevents High-Fat Diet-Induced Obesity in AKR/J Mice

**DOI:** 10.1371/journal.pone.0038734

**Published:** 2012-06-18

**Authors:** Yongjie Ma, Dexi Liu

**Affiliations:** Department of Pharmaceutical and Biomedical Sciences, College of Pharmacy, University of Georgia, Athens, Georgia, United States of America; National Institute of Agronomic Research, France

## Abstract

Pregnane X receptor (PXR) is known to function as a xenobiotic sensor to regulate xenobiotic metabolism through selective transcription of genes responsible for maintaining physiological homeostasis. Here we report that the activation of PXR by pregnenolone 16α-carbonitrile (PCN) in AKR/J mice can prevent the development of high-fat diet-induced obesity and insulin resistance. The beneficial effects of PCN treatment are seen with reduced lipogenesis and gluconeogenesis in the liver, and lack of hepatic accumulation of lipid and lipid storage in the adipose tissues. RT-PCR analysis of genes involved in gluconeogenesis, lipid metabolism and energy homeostasis reveal that PCN treatment on high-fat diet-fed mice reduces expression in the liver of *G6Pase*, *Pepck, Cyp7a1, Cd36, L-Fabp, Srebp*, and *Fas* genes and slightly enhances expression of *Cyp27a1* and *Abca1* genes. RT-PCR analysis of genes involved in adipocyte differentiation and lipid metabolism in white adipose tissue show that PCN treatment reduces expression of *Pparγ2, Acc1, Cd36*, but increases expression of *Cpt1b* and *Pparα* genes in mice fed with high-fat diet. Similarly, PCN treatment of animals on high-fat diet increases expression in brown adipose tissue of *Pparα*, *Hsl, Cpt1b*, and *Cd36* genes, but reduces expression of *Acc1* and *Scd-1* genes. PXR activation by PCN in high-fat diet fed mice also increases expression of genes involved in thermogenesis in brown adipose tissue including *Dio2, Pgc-1α, Pgc-1β*, *Cidea*, and *Ucp-3*. These results verify the important function of PXR in lipid and energy metabolism and suggest that PXR represents a novel therapeutic target for prevention and treatment of obesity and insulin resistance.

## Introduction

Pregnane X receptor (PXR; steroid and xenobiotic receptor or SXR) is a nuclear hormone receptor activated by xenobiotics as well as by diverse steroids and their metabolites [1]–[3]. PXR is known to induce, upon ligand binding, the expression of genes coding for phase I (Cyp3a11, CYP3A4, Cyp2b10) and phase II (Sult2a1, UDP-glucuronosyltransferase and GST) enzymes and drug transporters (MDR1) [4]. A good example of PXR-mediated regulation is transcription of *CYP3A4* gene encoding an enzyme responsible for the metabolism of more than 50% of clinical drugs [5].

**Figure 1:**
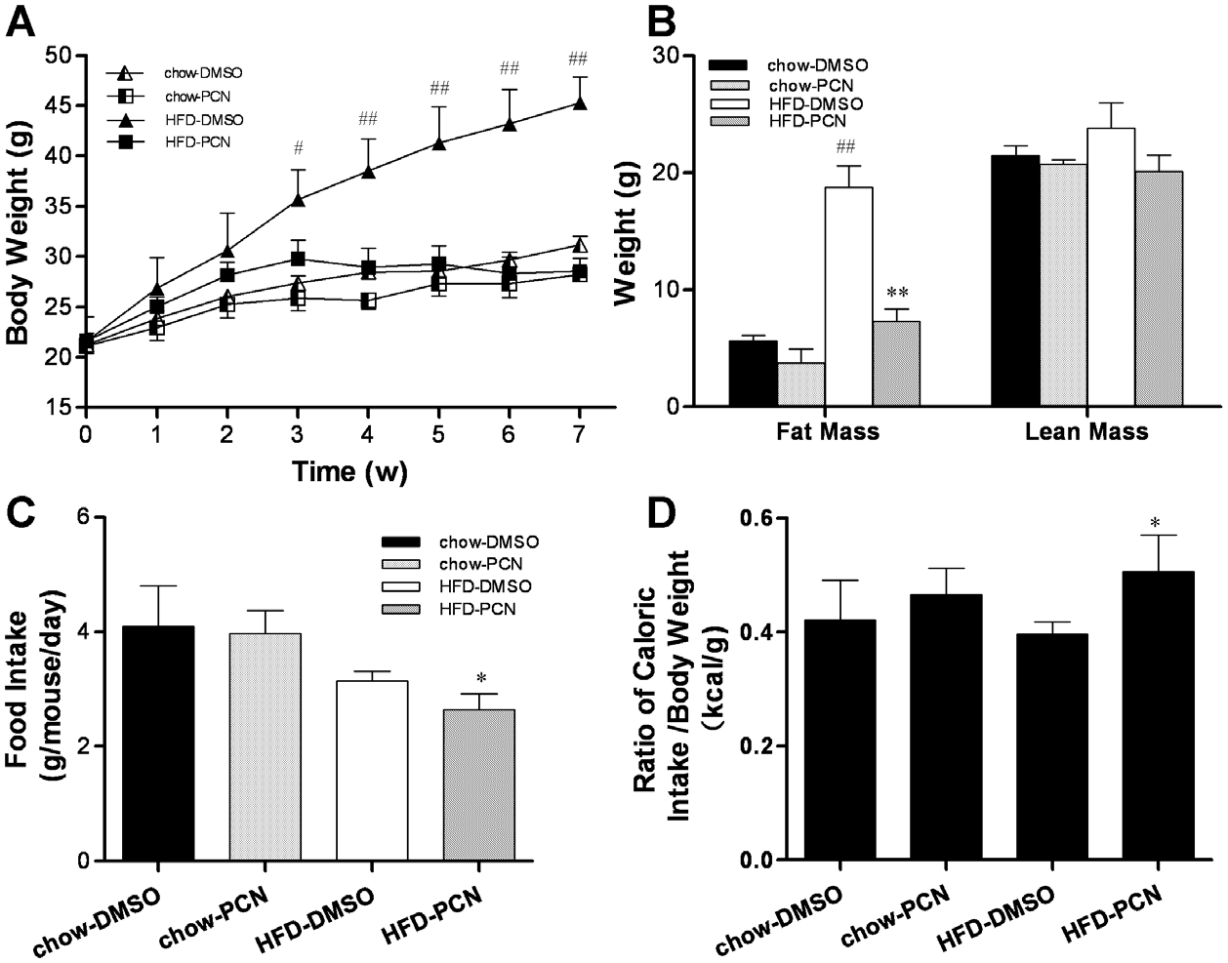
PCN treatment protected mice against high-fat diet–induced obesity. Four-week-old male AKR/J mice were fed with high-fat diet or regular chow for 7 weeks with twice weekly injections of PCN (50 mg/kg, IP) or DMSO (carrier solution). **A,** growth curve; **B**, fat and lean mass; **C,** food intake; and **D**, calculated ratio of caloric intake/body weight. Each data point represents the average ± SD of 4 animals in each group. #P<0.05, ##P<0.01 compared to chow-DMSO group; *P<0.05, **P<0.01 compared to high-fat diet-DMSO group.

In recent years, a number of clinical observations using PXR activators have linked PXR to lipid metabolism and energy homeostasis. Notably, treating with rifampicin, a PXR ligand, can influence lipid metabolism [6]. Similarly, treating children with antiepileptic drugs carbamazipine and phenobarbital for an extended time, could activate PXR and increase cholesterol levels [7]. Transgenic mice expressing constitutively activated PXR showed hepatic steatosis [8]. However, PXR also modulated sterol regulatory element binding protein 1 (SREBP-1) by inducing *Insig-1* expression, resulting in decreased levels of active SREBP-1 and reduced triglyceride synthesis [9]. Although additional studies are needed to resolve the seemingly contradictory effects of PXR activation in lipid homeostasis, the results from these studies firmly establish the role of PXR in regulating lipid and energy homeostasis at multiple levels.

Confirmation of the functional role of PXR in lipid metabolism has provided an opportunity to explore the mechanisms through which PXR agonists may impact energy homeostasis. Therefore, in this study, a mouse model was used to assess the effect of PXR activation on prevention of high-fat diet-induced obesity and insulin resistance. PXR activation was achieved by intraperitoneal injections of pregnenolone 16 α-carbonitrile (PCN), a mouse specific PXR activator. In AKR/J mice we demonstrate that PXR activation is capable of regulating lipid metabolism and energy expenditure, and consequently, preventing the development of high-fat diet-induced obesity and insulin resistance.

## Results

### PXR Activation Prevented Animals from Development of High-fat Diet-induced Obesity

To explore whether PXR plays an important role in the development of high-fat diet-induced obesity, 4-week old male AKR/J mice were fed a high-fat diet or regular chow as a control for 7 weeks, and simultaneously treated with PCN (50 mg/kg, twice weekly) or vehicle (DMSO). AKR/J mice are an obesity-prone inbred strain which gain body weight and fat more quickly compared to the C57BL/6J strain when fed with high-fat diet [10], [11]. They are also more insulin resistant [12] and therefore, are commonly used as a model for research on diet-induced obesity and obesity-related insulin resistance. As shown in Figure 1A, PCN treatment did not affect the growth rate of mice on regular chow. However, for animals fed with high-fat diet, PCN treatment resulted in a significant decrease in growth rate as compared to those treated with DMSO. A statistical difference was evidenced as early as the first 3 weeks of high-fat diet feeding. After 7 weeks, the average body weight of PCN treated animals was 28.6±1.3 g, 16.7 g less than the DMSO treated control groups at 45.3±2.5 g. There was no statistical difference between PCN-treated animals on high-fat diet and those on regular chow. The difference in body weight between DMSO-treated animals on a high-fat diet and the remaining animals is largely due to the difference in fat mass (Figure 1B). An approximately 60% reduction in fat mass was seen in PCN-treated animals fed with high-fat diet as compared to those of DMSO injected controls. There was no statistical difference in lean mass among animals fed with either regular chow or high-fat diet. When mice were fed with high-fat diet, the food intake per mouse per day in the PCN-treated group was lower when compared to DMSO-treated controls (Figure 1C). However, the caloric intake by PCN-treated animals appears slightly higher when corrected for total body weight (Figure 1D).

**Figure 2:**
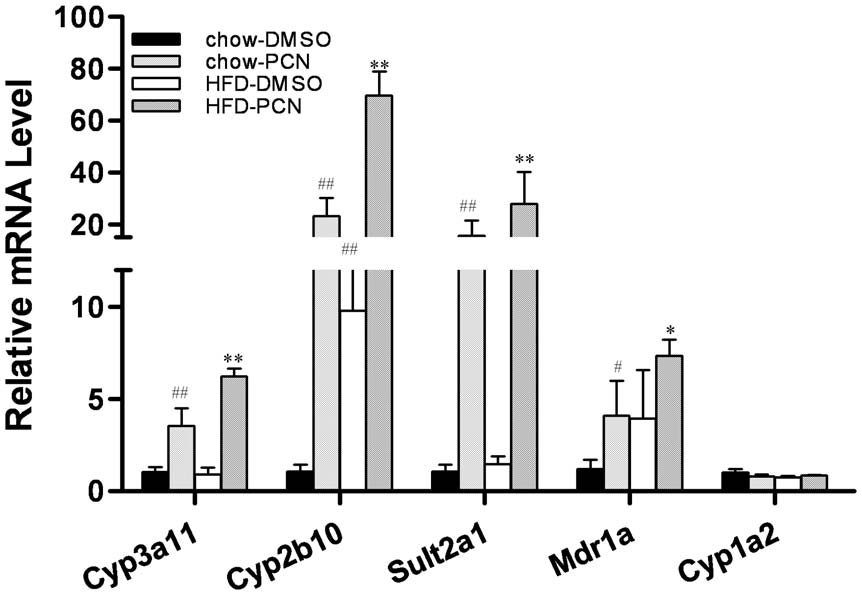
Up-regulation of PXR target genes by PCN treatment. At end of the 7-week treatment with PCN, mice were sacrificed 4 h later and livers were taken and frozen at -80°C. Hepatic expressions of selected genes were measured by real-time PCR analysis. #P<0.05, ##P<0.01 compared to chow-DMSO group; *P<0.05, **P<0.01 compared to high-fat diet-DMSO group. Abbreviations: *Cyp3a11*, cytochrome P450, family 3, subfamily a, polypeptide 11 gene; *Cyp2b10*, cytochrome P450, family 2, subfamily b, polypeptide 10 gene; *Sult2a1*, cytosolic sulfotransferase 2A1 gene; and *Mdr1a*, multi-drug-resistance 1a gene. Each data point represents the average ± SD of 4 animals in each group.

**Figure 3:**
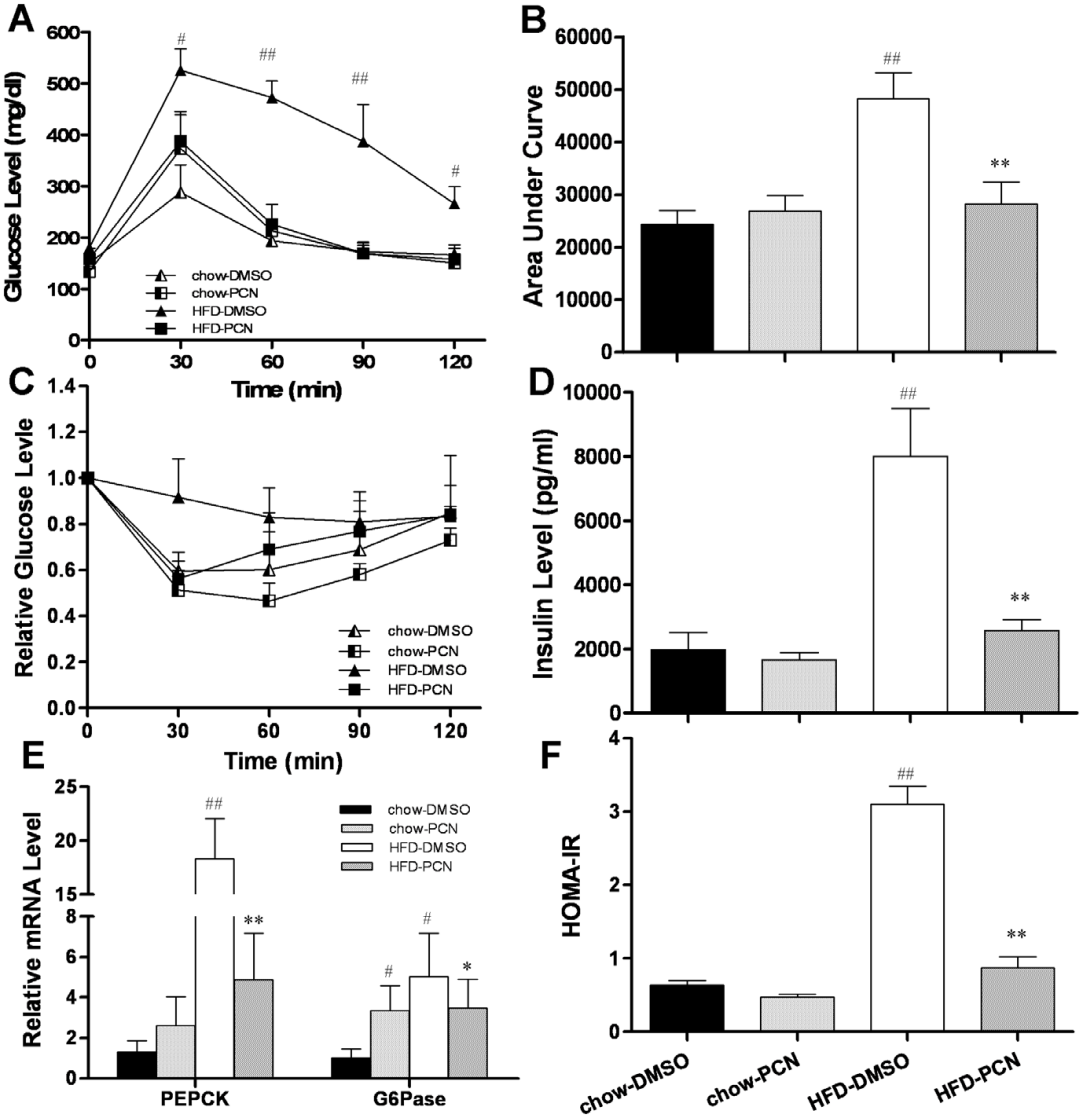
Effect of PCN treatment on glucose tolerance, insulin sensitivity, serum concentration of insulin, and mRNA level of *G6Pase* and *Pepck*. Animals at the end of the 7-week treatments were fasted overnight for glucose tolerance tests, or fasted for 4 h for insulin sensitivity tests. **A**, time-dependent blood concentration of glucose upon IP injection of glucose (2 g/kg); **B**, area under the curve from glucose tolerance test in A; **C**, time dependent glucose concentration upon IP injection of insulin; **D**, insulin levels at the end of the 7-week feeding with high-fat diet and regular chow food with or without PCN treatment; **E**, relative mRNA level of *G6Pase* and *Pepck* in mouse liver at the end of animal feeding and PCN treatment; and **F**, HOMA-IR values calculated based on formula: Glucose (mg/dl) × Insulin ( µU/ml)/405. Each data point represents the average ± SD of 4 animals in each group. #P<0.05, ##P<0.01 compared to chow-DMSO group; *P<0.05, **P<0.01 compared to high-fat diet-DMSO group.

In order to confirm whether the effects of PCN treatment seen in affected animals is correlated to PXR activation, the transcript levels of PXR target genes were measured. Results in Figure 2 show that PCN treatment significantly enhanced the expression of genes coding for enzymes involved in drug metabolism including *Cyp3a11, Cyp2b10*, *Sulat2a1* and *Mdr1a*, regardless of whether animals were on regular chow or on a high-fat diet. However, Figure 2 also showed that the high-fat diet is also capable of elevating gene expression for *Cyp3a11* and *Cyp2b10*. Since *CYP3A* and *CYP2B* genes can be regulated by both PXR and CAR [13], we included in the study of *Cyp1a2*, a target gene specific to CAR [14]. No increase in *Cyp1a2* mRNA level in PCN treated animals, whether on regular chow or a high-fat diet, suggests that PCN effect observed is mediated by PXR activation. This result agrees with the previous report that the mRNA level of *Cyp2b10* was elevated in genetic *ob/ob* male mice [15]. Considered together, these results demonstrated that PXR activation is responsible for protection animals against high-fat diet-induced obesity.

**Figure 4:**
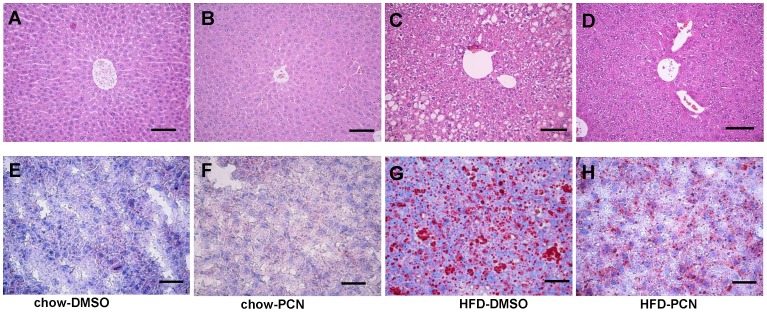
Effect of PCN treatment on hepatic accumulation of lipids. At the end of the 7-week treatment, animals were sacrificed; liver sections were made and stained with H&E (**A–D**) or Oil Red O (**E–H**). **A** and **E**, animals on regular chow and treated with DMSO; **B** and **F**, animals on regular chow and treated with PCN; **C** and **G,** animals on high-fat diet and treated with DMSO; and **D** and **H**; animals on high-fat diet and treated with PCN. Scale bar, 20 µm.

### PCN Treatment Improved Insulin Sensitivity of Animals Fed with High-fat Diet

Obesity is frequently associated with insulin resistance, a characteristic of type-2 diabetes. Next, we investigated whether PXR activation would affect glucose homeostasis. Glucose tolerance tests (Figure 3A) and calculated area under the curve (AUC) (Figure 3B) showed that there was no difference in glucose clearance among animals fed with regular chow and those fed with high-fat diet plus PCN treatment. The high-fat diet-fed control animals (treated with DMSO) exhibited a much slower clearance rate of intraperitoneally injected glucose. Protection against diet-induced insulin resistance was also confirmed using an insulin tolerance test (ITT). Results in Figure 3C show rapid reduction of glucose concentration in serum upon insulin injection in animals fed on regular chow or PCN treated animals fed with high-fat diet, as compared with DMSO-treated control on high-fat diet. Similar patterns were obtained in insulin level in serum (Figure 3D) and by HOMA-IR (Figure 3F). These results demonstrate that PCN treatment prevents the progression of insulin resistance in animals on high-fat diet.

**Figure 5:**
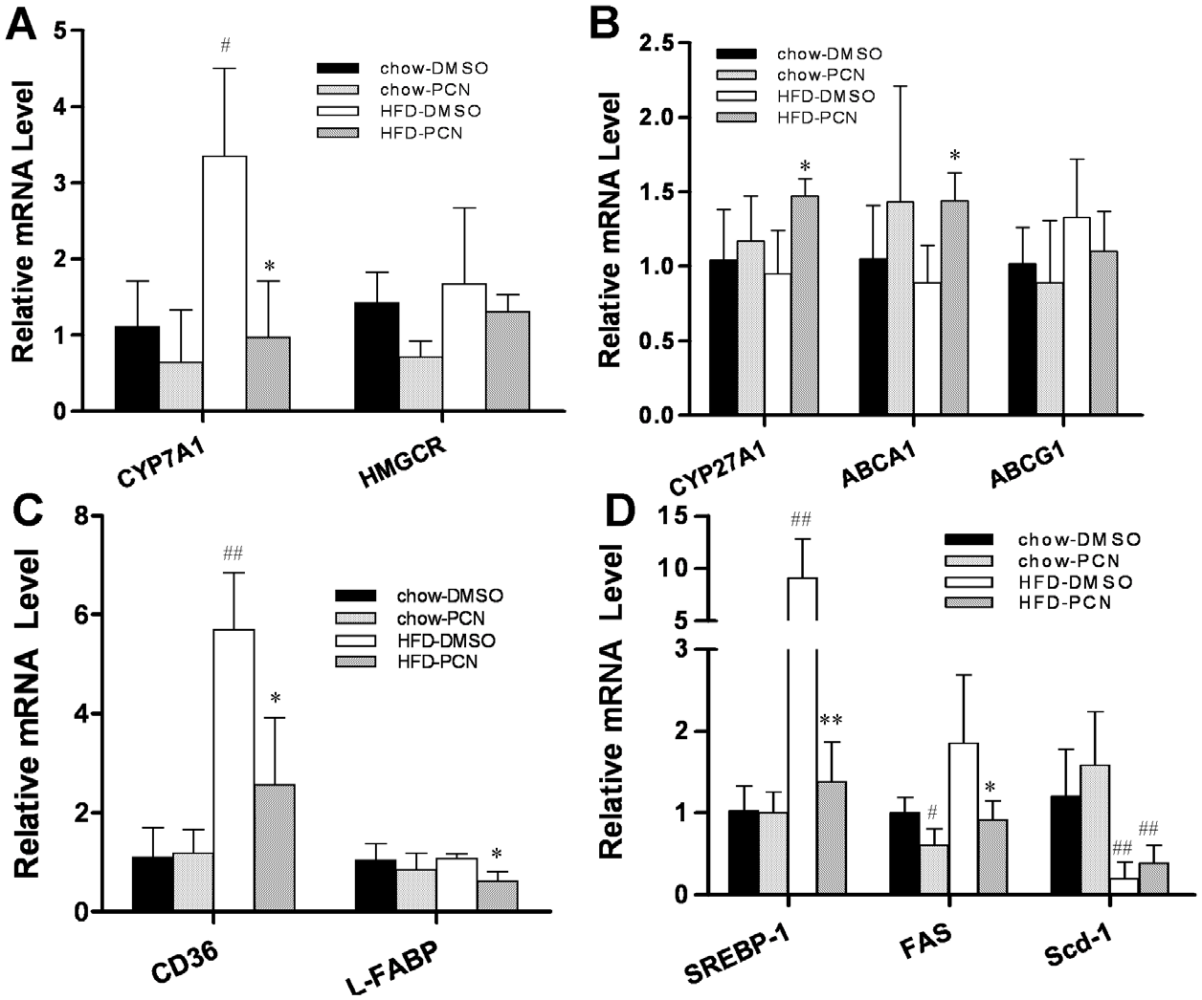
Effect of PCN treatment on expression of genes involved in lipogenesis and lipids uptake. Four h after treatment, mice were sacrificed and livers were harvested. The expression of genes involved in the cholesterol metabolism (**A**, **B**), lipids metabolism (**C**) and lipids uptake (**D**) were measured by real-time PCR. Each data point represents the average ± SD of 4 animals in each group. #P<0.05, ##P<0.01 compared to chow-DMSO group; *P<0.05, **P<0.01 compared to high-fat diet-DMSO group. Abbreviations: Cyp7a1, cholesterol 7 alpha-hydroxylase gene; HMGCR, 3-hydroxy-3-methylglutaryl coenzyme A reductase gene; Cyp27a1, sterol 27-hydroxylase; Abca1, Abcg1, ATP-binding cassette transporter A1 and G1; SREBP*-*1c, sterol regulatory element-binding protein 1c; FAS, fatty acid synthase; Scd-1, stearoyl CoA desaturase 1; and L-FABP, liver fatty-acid-binding protein.

**Figure 6:**
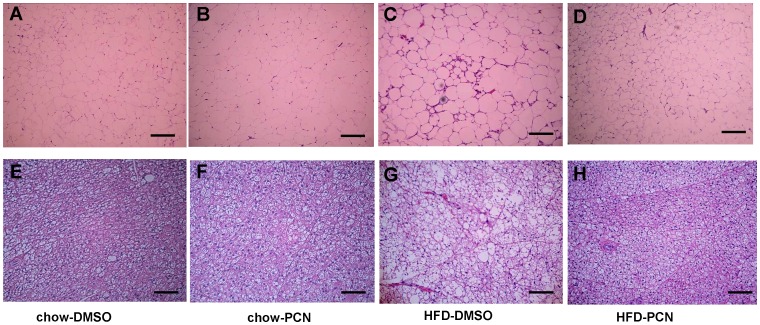
Effect of PCN treatement on lipid accumulation in adipose tissues. At the end of the 7-week treatment, animals were sacrificed, white (**A–D**) and brown (**E–H**) adipocyte tissues were collected and stained with H&E. **A** and **E**, animals on regular chow and treated with DMSO; **B** and **F**, animals on regular chow and treated with PCN; **C** and **G,** animals on high-fat diet and treated with DMSO; and **D** and **H**; animals on high-fat diet and treated with PCN. Scale bar, 20 µm.

To investigate how PCN treatments maintain the insulin sensitivity in animals fed with high-fat diet, we measured the expression of genes involved in hepatic gluconeogenesis after mice were sacrificed. Results (Figure 3E) from real time PCR reveal a marked increase in the amount of transcript for phosphoenolpyruvate carboxykinase (*Pepck*) and glucose-6-phosphatase (*G6Pase*) genes in the high-fat diet group treated with DMSO, concordant with hyperglycemia and hyperinsulinemia. Although PCN slightly increased the expression levels of both genes in mice fed with regular food, PCN treatment had a significantly lower high-fat diet-induced increase in gene expression of *Pepck* and *G6Pase* by 73% and 31%, respectively. It is noteworthy that the *Pepck* promoter contains multiple transcription factor binding sites for rapid transcription regulation [16]. In contrast, further studies are needed to demonstrate whether PCN plays a role in regulating the activity of PEPCK enzymes. Overall, these results suggest that inhibition of high-fat diet-induced gluconeogenesis by PCN is responsible, at least in part, for maintaining insulin sensitivity.

**Figure 7:**
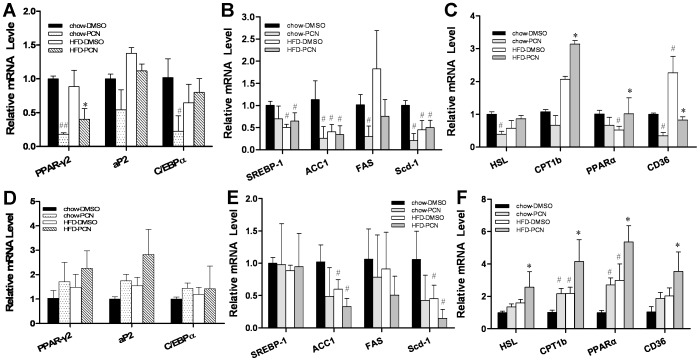
Effect of PCN treatment on expression of genes involved in adipocyte differentiation and lipid metabolism. At the end of the 7-week treatment animals were treated with PCN or DMSO (carrier solution) and sacrificed 4 h later. WAT and BAT tissues were harvested and total RNA was extracted. Relative mRNA level of genes involved in adipose differentiation in WAT (**A**) and BAT (**D**) tissue; genes involved in the lipogenesis in WAT (**B**) and BAT (**E**); and genes involved in lipids uptake and β-oxidation in WAT (**C**) and BAT (**F**)**.** Each data point represents the average ± SD of 4 animals in each group. #P<0.05, ##P<0.01 compared to chow-DMSO group; *P<0.05, **P<0.01 compared to high-fat diet-DMSO group. Abbreviations: aP2, adipocyte protein 2 gene; C*/*EBPα, CCAAT/enhancer binding protein alpha gene; PPARγ, peroxisome proliferator-activated receptor gamma gene; ACC1, acetyl-CoA carboxylases 1 gene; CPT1b, carnitine palmitoyltransferase 1b gene; and HSL, hormone-sensitive lipase gene.

**Figure 8:**
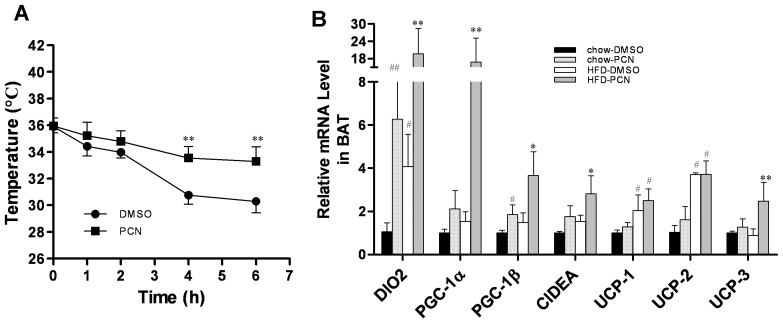
Effect of PCN treatment on energy expenditure of brown adipose tissue. Six-week-old male AKR/J mice were fed with high-fat diet for two weeks with three injections per week of PCN (50 mg/kg) or DMSO (control). By the end of the feeding period, mice were exposed at 4°C and the rectal temperature measured at different times. **A**, time-dependent rectal temperature. For determination of the relative mRNA level of genes involved in thermogenesis, animals at the end of 7-week treatment were injected with PCN or carrier solution and sacrificed 4 h later. BAT tissues were harvested and total RNA was extracted. **B.** Relative mRNA levels of genes involved in thermogenesis. Each data point represents the average ± SD of 4 animals in each group. #P<0.05, ##P<0.01 compared to chow-DMSO group; *P<0.05, **P<0.01 compared to high-fat diet-DMSO group. Abbreviations: PGC-1, peroxisome proliferator-activated receptor gamma coactivator 1 gene; Dio2, Type II iodothyronine deiodinase gene; Cidea, cell death-inducing DNA fragmentation factor alpha-like effector A gene; and UCP, uncoupling protein gene.

**Table 1:** Primer sets for real time RT-PCR analysis of gene expression.

Name	Forward sequence	Reverse sequence
*Cyp3a11*	CGCCTCTCCTTGCTGTCACA	CTTTGCCTTCTGCCTCAAGT
*Cyp2b10*	TCCTGACCAGTTCCTGGATG	CTGGAGGATGGACGTGAAGAA
*Sult2a1*	AGGACCACGACTCATAACCTCCCA	CCGAGTGACCCTGGATTCTTCACA
*Mdrla*	CAGCAGTCAGTGTGCTTACAA	ATGGCTCTTTTATCGGCCTCA
*Pepck*	AAGCATTCAACGCCAGGTTC	GGGCGAGTCTGTCAGTTCAAT
*G6Pase*	CGACTCGCTATCTCCAAGTGA	GTTGAACCAGTCTCCGACCA
*Cyp7a1*	AACGGGTTGATTCCATACCTGG	GTGGACATATTTCCCCATCAGTT
*Hmgcr*	CTTGTGGAATGCCTTGTGATTG	AGCCGAAGCAGCACATGAT
*Cyp27a1*	GACAACCTCCTTTGGGACTTAC	GTGGTCTCTTATTGGGTACTTGC
*Abca1*	AAAACCGCAGACATCCTTCAG	CATACCGAAACTCGTTCACCC
*Abcg1*	GCTCCATCGTCTGTACCATCC	TGTTCTGATCCCCGTACTCCC
*Srbp-1*	CCCTGTGTGTACTGGCCTTT	TTGCGATGTCTCCAGAAGTG
*Acc-1*	GCCTCTTCCTGACAAACGAG	TGACTGCCGAAACATCTCTG
*Fas*	AGAGATCCCGAGACGCTTCT	GCCTGGTAGGCATTCTGTAGT
*Scd-1*	TTCTTACACGACCACCACCA	CCGAAGAGGCAGGTGTAGAG
*Cd36*	CCTTAAAGGAATCCCCGTGT	TGCATTTGCCAATGTCTAGC
*L-Fabp*	ATGAACTTCTCCGGCAAGTACC	GTGACACCCCCTTGATGTCC
*Pparγ2*	TCG CTG ATG CAC TGC CTA TG	GAG AGG TCC ACA GAG CTG ATT
*aP2*	AAGGTGAAGAGCATCATAACCCT	TCACGCCTTTCATAACACATTCC
*C/Ebpα*	CAAGAACAGCAACGAGTACCG	GTCACTGGTCAACTCCAGCAC
*Hsl*	GCTTGGTTCAACTGGAGAGC	GCCTAGTGCCTTCTGGTCTG
*Cpt1b*	CTCCGCCTGAGCCATGAAG	CACCAGTGATGATGCCATTCT
*Pparα*	TGTCGAATATGTGGGGACAA	AATCTTGCAGCTCCGATCAC
*Dio2*	AATTATGCCTCGGAGAAGACCG	GGCAGTTGCCTAGTGAAAGGT
*Pgc-1^α^*	GAAGTGGTGTAGCGACCAATC	AATGAGGGCAATCCGTCTTCA
*Pgc-1β*	TTGTAGAGTGCCAGGTGCTG	GATGAGGGAAGGGACTCCTC
*Cidea*	ATCACAACTGGCCTGGTTACG	TACTACCCGGTGTCCATTTCT
*Ucp-1*	AGGCTTCCAGTACCATTAGGT	CTGAGTGAGGCAAAGCTGATTT
*Ucp-2*	GCGTTCTGGGTACCATCCTA	GCTCTGAGCCCTTGGTGTAG
*Ucp-3*	ATGAGTTTTGCCTCCATTCG	GGCGTATCATGGCTTGAAAT
*Gapdh* *Cyp1a2*	AGGTCGGTGTGAACGGATTTG ATGAGGAGCTGGACACGGTG	TGTAGACCATGTAGTTGAGGTCA TCCACTGCTTCTCATCATGG

### Inhibition of high-fat diet-induced lipid accumulation by PCN treatment

Hepatic lipid accumulation has a strong correlation with obesity and insulin resistance [17]. We further evaluated the effect of PCN on hepatic cholesterol and lipid metabolism. H&E stained liver sections showed a normal liver structure of mice on regular chow with or without PCN treatment (Figures 4A and 4B). However, there was extensive hepatocyte vacuolation in animals fed with a high-fat diet (Figure 4C), reflecting intrahepatic fat accumulation in high-fat diet-fed mice. In contrast, PCN treatment efficiently ameliorated lipids accumulation in hepatocytes (Figure 4D). Oil Red O staining of liver sections confirmed no oil accumulation in animals on regular chow (Figures E and F), but a massive accumulation of fatty components in the livers of mice on a high-fat diet (Figure 4G), and significantly less oil accumulation in the PCN-treated group (Figure 4H).

To determine how PCN reduces the excess lipid accumulation in the liver of animals on a high-fat diet, we performed analyses on the expression profiles of genes that are involved in cholesterol and lipids metabolism. As shown in Figure 5A, PCN treatment inhibited the cholesterol 7 α-hydroxylase (*Cyp7a1*) gene expression on regular chow and on high-fat diet, in agreement with the results from previous studies [18], [19]. PCN did not affect the expression of the 3-hydroxy-3-methylglutaryl coenzyme A reductase (*Hmgcr*) gene, a key enzyme in cholesterol biosynthesis. Compared to animals on regular chow, PCN treatment of animals on high-fat diet elevated mRNA levels of sterol 27-hydroxylase (*Cyp27a1*) and ATP-binding cassette transporter *Abca1* (Figure 5B), the genes involving cholesterol efflux. PCN treatment slightly reduced transcript levels of the *Abcg1* gene.

The most striking changes were seen in genes that regulate lipogenesis and lipid uptake. *CD36* is responsible for the transport of long-chain fatty acids into the adipose and hepatic tissues [20]–[22]. Increased hepatic *CD36* activity is critical for the development of steatosis in obesity [22]–[24]. Results in Figure 5C showed that *Cd36* was up-regulated 5.7-fold in high-fat diet-fed mice compared to that of mice on regular food. However, PCN treatment attenuated the high-fat diet-induced transcription of *Cd36* by 55%. At the same time, PCN also decreased the mRNA level of liver fatty acid binding protein (L-FABP) when mice were fed with a high-fat diet. PCN treatment significantly inhibited high-fat diet-induced increase of *Srebp*-*1c* gene expression and its target gene responsible for fatty acid synthase *(Fas*) by 85% and 50%, respectively. Both SREBP*-1c* and FAS are key regulatory enzymes in the lipogenetic pathway (Figure 5D). These data suggested that decreased hepatic lipogenesis and fatty acid uptake could alleviate high-fat diet-induced hepatic fat accumulation.

### PCN Treatment Prevented Adiposity in White Adipose Tissue (WAT) and Brown Adipose Tissue (BAT) of Animals Fed with High-fat Diet

Excessive deposition of lipids in adipose tissues is one of the major characteristics in obesity. Comparing to that of animals on regular chow (Figures 6A and 6B), the size of adipocytes in WAT of high-fat diet-fed animals is significantly larger (Figure 6C). However, there is no difference between animals on a regular chow (Figures 6A and 6B) and those treated with PCN when on a high-fat diet (Figure 6D). Reduction of lipids accumulation is even more prominent in BAT. Larger adipocytes are evidenced in animals on a high-fat diet (Figure 6G) as compared to those of animals either on regular chow (Figures 6E and 6F) or treated with PCN when on high-fat diet (Figure 6H).

Quantitative PCR was performed on total RNAs extracted from WAT and BAT to examine the PCN effect on transcription of adipocyte protein 2 (aP2), CCAAT/enhancer binding protein α (*C*/*Ebp*α) and *Ppar*γ2 gene in high-fat diet-fed mice. Results in Figure 7A show that in WAT, PCN reduces the expression of *Ppar*γ2. PCN induces a significant reduction in mRNA level of C*/*EBPα in animals fed with regular chow but not in high-fat diet-fed animals. Previous studies indicated that PXR is a positive regulator of *PPARγ* in the liver [25]. However, *PPAR*γ2 is mostly expressed in the adipose tissue [26]. Further study is needed to elucidate the mechanism of the differential regulation. PCN also did not alter the expression of the same set of genes in BAT (Figure 7D), suggesting that the reduction in total fat mass may be a result from less triglyceride accumulation rather than a reduced number of adipocytes. At the same time, high-fat diet feeding did not induce lipogenesis in WAT and BAT (Figures 7B, 7E). However, lipolysis and β-oxidization is significantly enhanced by PCN in high-fat diet-fed mice, as evidenced by elevation of gene expression of *Pparα*, *Cpt1b* and *Hsl* in WAT and BAT (Figures 7C, 7F). In WAT, PCN lowered the high-fat diet-induced CD36 expression by 60%. In contrast, PCN up-regulated the mRNA level of *Cd36* in BAT, suggesting difference between WAT and BAT in lipid metabolism.

### PCN Treatment Increased Expression of Genes Involved in Energy Expenditure in High-fat Diet-fed Mice

BAT plays an important role in thermogenesis [27]–[29]. To further analyze the potential mechanisms of PCN effect, we measured the changes of body temperature when these animals were exposed to 4°C. Results in Figure 8A show that mice treated with PCN had a much slower decrease in body temperature than the DMSO-treated control, suggesting that activation of PXR enhanced the thermogenic activity in BAT. At the molecular level, PCN treatment significantly enhanced the transcription of genes that are critical for cellular thermogenesis including *Dio2, Pgc-1α, Pgc-1β, Cidea*, and *Ucp-3*. No difference was seen in *Ucp-2* mRNA level of high-fat diet-fed animals with or without PCN treatment (Figure 8B). These results suggest that PCN treatment increased energy expenditure in BAT.

## Discussion

In this study, we demonstrate that activation of PXR by PCN prevented development of high-fat diet-induced obesity and relieved obesity-related insulin resistance and hepatic lipid accumulation (Figures 1, 3, 4). The beneficial effect of PCN treatment was achieved by inhibition of lipogenesis and gluconeogenesis in the liver (Figures 3E and 5) as well as inhibition of lipid uptake in liver and WAT (Figures 5c and 7c), by enhanced lipolysis in adipose tissue (Figures 7C, 7F), and by increased peripheral fat mobilization and energy expenditure in BAT (Figure 8).

The hypoglycemic effect after PCN treatment on animals on high-fat diet was mediated by inhibiting diet-induced increase of PEPCK and G6Pase (Figure 3E), two key enzymes in gluconeogenesis, in agreement with previous reports [30]. It is possible that activated PXR directly interacts with FoxO1, one of the transcription factors that plays a critical role in lipid metabolism and gluconeogenesis in the liver [31], and prevents FoxO1 from binding to IRS, resulting in the suppression of *G6Pase* and *Pepck1* gene expression [32]. In addition, inhibition of lipid accumulation by PCN may also contribute indirectly in maintenance of glucose homeostasis because lipotoxicity in the liver and pancreas exerts an important function in type-2 diabetes [33]. It is still unknown, however, how PCN improves obesity-induced insulin resistance. Decrease in fat accumulation in WAT may benefit improved insulin sensitivity. It’s well known that adipocytes in WAT release significant amounts of pro-inflammatory cytokines such as TNFα, IL-6 and IL-1. Obesity also resulted in more macrophage infiltration in adipose tissue [34]. These pro-inflammatory cytokines and chronic inflammation in fat are major factors in causing the whole-body obesity-related insulin resistance [35]. Our unpublished data show that PCN decreases macrophage accumulation and pro-inflammatory cytokine release in adipose tissue. In addition, the NFκB pathway plays a crucial role in production of pro-inflammatory cytokines. While activation of PXR inhibits the activity of NFκB [36], PCN may increase insulin sensitivity through inhibiting obesity-induced chronic inflammation.

Results shown in Figures 4 and 6 demonstrate that PCN-mediated PXR activation significantly prevented the lipid storage in adipose tissue and the liver. Except for inhibiting lipogenesis, a more important characteristic of PXR in diet-induced obesity was reduction of lipid uptake in liver and adipose, confirmed by inhibiting up-regulation of *Cd36* expression by high-fat diet. *Cd36* codes for a scavenger receptor with broad ligand specificity. Activation of *Cd36* facilitates free fatty acid uptake from circulation and also contributes to obesity, hepatic steatosis and type-2 diabetes [24], [37]. Previous studies show that *Cd36* is a shared transcriptional target of LXR, PXR and PPARγ in their regulation of lipid homeostasis [25]. In the PXR-transgenic mice, *Cd36* was up-regulated when fed with regular chow, which is different from our result obtained from the high-fat diet-fed mice. In our opinion, as in drug metabolism, PXR may serve as a “sensor” for maintaining energy homeostasis.

Unlike the effect of PCN in the liver and WAT, PCN treatment increased *Cd36* transcript level by approximately 2-fold compared to the DMSO-treated group in BAT (Figure 7F). Accumulation of lipids induced by a high-fat diet was blunted, indicating another mechanism existed in BAT. The main function of BAT is to generate heat for thermogenesis. Bartelt *et al.* [38] reported that increased BAT activity enhanced triglyceride-rich lipoprotein metabolism in mice. Exposure to low temperatures drastically accelerated plasma clearance of triglycerides as a result of increased uptake into BAT, which was mediated by an increase of transmembrane receptor CD36. Although it remains to be demonstrated for increased energy expenditure, mice with PCN treatment had a much slower decrease in body temperature than the control group (Figure 8A), suggesting that the activation of PXR enhanced the thermogenesis activity in BAT. At the same time, data from real-time PCR showed a significant increase in the transcription of genes involved in fatty acid β-oxidization (Figure 7F) and thermogenesis (Figure 8B), indicating increased energy expenditure in BAT, which contributed to cleaning more lipids in BAT.

In summary, our study demonstrates that PCN-mediated PXR activation prevented diet-induced obesity in AKR/J mice, decreased lipid accumulation and maintained insulin sensitivity. Additional work is needed to illustrate the precise mechanisms through which PXR modulates energy metabolism and lipid homeostasis. The effect of chronic activation of PXR on regulating inflammation, another important factor associated with obesity and type-2 diabetes, deserves attention. Considering that obesity has become an important health problem in the recent century with an estimated one billion people overweight and at least 300 million obese adults in the world [39], our results suggest PXR may represent a novel therapeutic target for prevention and treatment of obesity and type-2 diabetes. As PCN is a specific activator for mouse PXR, caution should be taken when extending the current conclusions to humans.

## Materials and Methods

### Materials

Pregnenolone-16α-carbonitrile was purchased from Sigma (St. Louis, MO). High-fat diet (60% kJ/fat, 20% kJ/carbohydrate, 20% kJ/protein) was purchased from Bio-serv (Frenchtown, NJ,catalog number S3282). RNeasy Tissue kit was from Qiagen (Valencia, CA). The SuperScript® III First-Strand Synthesis System was purchased from Invitrogen (Carlsbad, CA). Real-time PCR reagents were acquired from Applied Biosystems (Foster City, CA). An insulin assay kit was obtained from Crystal Chem (Downers Grove, IL). Oil Red O solution was obtained from Electron Microscopy Science (Hatfield PA). Glucometer and test strips were purchased from LifeScan (Milpitas, CA). AKR/J mice were purchased from the Jackson Laboratory (Bar Harbor, ME).

### Animals and Treatment

All procedures performed on mice were approved by the Institutional Animal Care and Use Committee at the University of Georgia, Athens, Georgia. Four-week-old male AKR/J mice were fed with high-fat diet or regular chow and received twice weekly injections of PCN (50 mg/kg) intra-peritoneally or DMSO (carrier solution) for 7 weeks. Animals were weighed weekly and their body composition was determined using EchoMRI-100TM from Echo Medical Systems (Houston, TX).

### Analysis of Serum Insulin Level

Blood samples were collected from fasted mice. Insulin level in the serum was measured using commercial assay kits according to the manufacturer’s instructions. HOMA-IR was calculated as: (fasting insulin [mU/ml] × fasting glucose [mg/dl])/405.

### Glucose Tolerance Test (GTT) and Insulin Tolerance Test (ITT)

For GTT, mice were injected intraperitoneally with glucose at 2 g/kg body weight after fasting overnight. Blood samples were taken at varying time points and the glucose concentrations were measured using a glucometer. For ITT, mice fasted for 4 h and blood glucose levels were measured after an intraperitoneal injection of human insulin (Novolin) from Novo Nordisk (Princeton, NJ) at 1.2 U/kg.

### Histochemical Analysis

After mice were sacrificed, the liver and white and brown adipose tissues were collected, fixed in 10% formalin, embedded in paraffin, and sectioned at a thickness of 5 µm. H&E staining was performed. Frozen sections (9 µm) were used for Oil Red O staining. Microscopic examination was performed and photographs were taken under a regular light microscope.

### Gene Expression Analysis by Real Time PCR

Total RNA was isolated from the mouse liver and white and brown adipose tissues using an RNeasy kit. Two µg of total RNA were used for the first strand cDNA synthesis as recommended by the manufacturer (Invitrogen, Carlsbad, CA). RT-PCR was performed using SYBR Green as an indicator on the ABI StepOne Plus Real-Time PCR system. The final reaction mixture contained 20 ng of cDNA, 250 nM of each primer, 10 µl of 2x SYBR Green PCR Master, and RNase-free water to complete the reaction mixture volume to 20 µl. The PCR was carried out for 40 cycles at 95°C for 15 s and 60°C for 1 min. Fluorescence was read during the reaction, allowing a continuous monitoring of the amount of PCR product. The data were normalized to internal control GADPH mRNA. The primer sequences employed are summarized in Table 1.

### Statistical Analysis

Statistical analysis was done by one-way ANOVA. All data are reported as mean ± standard deviation (SD) with statistical significance set at P<0.05.
